# Ventilação e Parâmetros Respiratórios em RCP: Onde Estamos e Próximos Passos!

**DOI:** 10.36660/abc.20230492

**Published:** 2023-09-04

**Authors:** Hélio Penna Guimarães

**Affiliations:** 1 Hospital Israelita Albert Einstein Universidade Federal de São Paulo São Paulo SP Brasil Hospital Israelita Albert Einstein / Universidade Federal de São Paulo-UNIFESP, São Paulo, SP – Brasil

**Keywords:** Reanimação Cardiopulmonar, Parada Cardíaca, Ventilação

“*Então formou o Senhor Deus o homem do pó da terra, e lhe soprou nas narinas o fôlego da vida, e o homem passou a ser alma vivente*”

Bíblia; Gênesis, 2:7.

A primeira menção de ressuscitação cardiopulmonar refere-se ao momento da criação de Adão, tendo Deus “soprado em sua boca, dando-lhe a vida”. Menos simbólica e mais precisa em seu detalhamento, e considerada por muitos historiadores como o primeiro relato de manobras de RCP, está também na bíblia a descrição no livro dos Reis sobre o profeta Eliseu, um discípulo de Elias, que reanimou um jovem filho de uma viúva sunamita. “...subiu à cama, deitou-se sobre o menino e, pondo a sua boca sobre a boca dele, os seus olhos sobre os olhos dele e suas mãos sobre as mãos dele, se estendeu sobre o menino; este espirrou sete vezes e abriu os olhos” manobra realizada por Eliseu, narrada no II Reis 4:34-35141.

Paracelsus avaliou, em 1530, uso de foles de lareira para introdução do ar nos pulmões de indivíduos aparentemente mortos, caracterizando as primeiras e rústicas tentativas de ventilação artificial, baseadas no princípio, até hoje utilizado, da ventilação sob pressão positiva, utilizando as unidades bolsa-valva-máscara.^1^

Tais relatos históricos têm em comum, desde as primeiras tentativas de ressuscitação cardiopulmonar (RCP), a preocupação em ventilar/promover a respiração, e não apenas a circulação, sempre foi fator de atenção para as manobras de suporte básico e avançado de vida.^2^

Após a fase inicial da RCP com prioridade de compressões utilizando a oxigenação residual sanguínea, a ventilação é essencial para garantir a oxigenação e eliminação de CO_2_.^2^

De fato, a maioria dos estudos sobre ventilação durante a RCP avalia sua interação com a eficácia das compressões torácicas e consequências hemodinâmicas. Os efeitos fisiológicos da ventilação durante a RCP ainda demandam de adequada compreensão abrangente de seu papel.

Com o avanço de conhecimentos e diretrizes em RCP, a ressuscitação cardiopulmonar (RCP) foi direcionada para a uma maior preocupação com qualidade de compressão, a despeito de que a ventilação adequada para evitar a hiperventilação continua sendo um problema comum, mesmo quando a RCP é realizada por equipes de ressuscitação adequadamente capacitadas. Isso faz com que o monitoramento da ventilação seja considerado um procedimento fundamental, a fim de evitar efeitos hemodinâmicos deletérios e o aumento das frequências respiratórias e dos volumes correntes causadores de barotrauma, volutrauma e atelectrauma, incorrendo em desfechos impactantes de morbidade e mortalidade nas síndromes pós RCP.^2^

Cordioli et al.^3^ registraram episódios de ventilação reduzida associada a compressões, retratando traçado de fluxo inspiratório limitado durante a descompressão torácica em PEEP zero. Estes autores justificam tal fato pela redução do volume pulmonar abaixo do volume torácico expiratório final induzido por compressões torácicas, podendo provocar o fechamento das vias aéreas distais e levar à limitação do fluxo.

Em modelos animais, o declínio ao longo do tempo de ventilação produzida apenas por compressões torácicas sozinhas levou a grandes áreas de atelectasias e menor PaO2 em comparação com animais ativamente ventilados precocemente durante a RCP. A via aérea mantida sobre pressão positiva permitiu a manutenção de uma ventilação eficiente e troca gasosa adequada apenas com compressões torácicas. A despeito disso, há um potencial efeito negativo da PEEP no fluxo sanguíneo durante a RCP que potencialmente prejudicaria o retorno venoso.^4,5^

Neste cenário, buscando estabelecer estratégias de ventilação prática e protetora durante a RCP, a ressuscitação cardiopulmonar e cerebral se apresenta como um desafio para os estudiosos.

Nesta edição, Palácio et al.^6^ avaliaram um ventilador mecânico portátil com pico de pressão inspiratória em modelo experimental com porcos com o intuito de avaliar a viabilidade da ventilação durante a RCP e comparar os parâmetros monitorados com a ventilação bolsa-válvula. Os autores demostraram taxas de retorno para circulação espontânea e saturação arterial de oxigênio semelhantes; com variações estatisticamente significativas de volume corrente, ETCO_2_ e pico de fluxo inspiratório, sendo menor a complacência pulmonar dinâmica após RCE no grupo bolsa-válvula. O artigo conclui que a ventilação *VLP2000E* é viável durante a RCP e equivalente à ventilação bolsa-válvula quanto às taxas de RCE e à saturação arterial de oxigênio, porém com melhor perfil de parâmetros respiratórios e menor pressão nas vias aéreas e volume corrente.

Considerando futuros e distintos cenários como ambiente pré-hospitalar ou mesmo departamentos de emergência, dispositivos com esta performance podem otimizar a capacidade de ventilação durante a RCP, reduzindo a necessidade de membros adicionais na equipe com ênfase na ventilação e permitindo maior foco de atenção a compressões torácicas, sem a perda da qualidade de ventilação e oxigenação. Estudos de ressuscitação cardiopulmonar são muito bem-vindos à literatura científica brasileira e a translação dos estudos pré-clínicos para estudos de fase III, visando a adequada avaliação dos achados, confere o próximo importante passo, similar aos dispositivos de feedback e compressão torácica, para efetivamente recompor a qualidade da ventilação com menores impactos de lesão pulmonar e período pós-PCR.
