# Supplementary material for: Ventilação e Parâmetros Respiratórios em RCP: Onde Estamos e Próximos Passos!
Source: Arq Bras Cardiol. 2023 Sep 4;120(7):e20230492. [Article in Portuguese] doi: 10.36660/abc.20230492 (PMC10519351; doi:10.36660/abc.20230492)
Supplement: Translation

# Translation: Ventilation and Respiratory Parameters in CPR: Where are We and Next Steps!

**Authors:** Hélio Penna Guimarães

**Affiliations:** 1 Brazil Hospital Israelita Albert Einstein / Universidade Federal de São Paulo-UNIFESP, São Paulo, SP – Brazil

**Keywords:** Cardiopulmonary Resuscitation, Heart Arrest, Ventilation

“*Then the Lord God formed Man from the dust of the ground, and breathed into his nostrils the breath of life, and man became a living soul.*”

Bible; Genesis, 2:7.

The first mention of cardiopulmonary resuscitation refers to the moment of Adam’s creation, in which God “breathed into his mouth, giving him life.” The Bible also mentions, in the book of the Kings, prophet Elisha, a disciple of Elijah, who revived a young son of a Shunamite widow, whose description is less symbolic and more precise in its detailing, being considered by many historians to be the first account of CPR maneuvers. “... and he went up, and lay upon the child, and put his mouth upon his mouth, and his eyes upon his eyes, and his hands upon his hands: and he stretched himself upon the child; the child sneezed seven times, and the child opened his eyes,” maneuver performed by Elisha, narrated in II Kings 4:34-35141.

Paracelsus evaluated, in 1530, the use of fireplace bellows to introduce air into the lungs of apparently dead individuals, characterizing the first and rustic attempts at artificial ventilation, based on the current principle of positive pressure ventilation using the bag-valve-mask units.^1^

These historical reports of cardiopulmonary resuscitation (CPR) attempts have one thing in common: the concern to ventilate/promote breathing and not only promote circulation has always been a factor of attention for basic and advanced life support maneuvers.^2^

After the initial phase of CPR with priority compressions using residual blood oxygenation, ventilation is essential to ensure oxygenation and elimination of CO_2_.^2^

In fact, most studies on ventilation during CPR evaluate its interaction with the efficacy of chest compressions and hemodynamic consequences. The physiological effects of ventilation during CPR still demand an adequate comprehensive understanding of its role.

With the advancement of knowledge and guidelines in CPR, cardiopulmonary resuscitation (CPR) was directed to a greater concern with compression quality, despite the fact that adequate ventilation to avoid hyperventilation remains a common problem, even when CPR is performed by adequately trained resuscitation teams. This requires ventilation monitoring to be considered a fundamental procedure, in order to avoid deleterious hemodynamic effects and the increase in respiratory rates and tidal volumes, which result in barotrauma, volutrauma and atelectrauma, incurring impacting morbidity and mortality outcomes in post-CPR syndromes.^2^

Cordioli et al.^3^ recorded episodes of reduced ventilation associated with compressions, depicting limited inspiratory flow tracing during thoracic decompression in PEEP zero. These authors attribute this fact to the reduction of lung volume below the end-expiratory thoracic volume induced by chest compressions, which can cause closure of the distal airways and lead to flow limitation.

In animal models, the decline over ventilation time produced only by chest compressions alone led to large atelectasis areas and lower PaO2 compared to animals that were ventilated early during CPR. The airway maintained under positive pressure allowed the maintenance of efficient ventilation and adequate gas exchange only with chest compressions. Despite these findings, there is a potential negative effect of PEEP on blood flow during CPR that would potentially impair venous return.^4,5^

In this scenario, seeking to establish strategies for practical and protective ventilation during CPR, cardiopulmonary and cerebral resuscitation poses itself as a challenge to scholars.

In this edition, Palacio et al.^6^ evaluated a portable mechanical ventilator with peak inspiratory pressure in an experimental model with pigs, aiming to evaluate the viability of ventilation during CPR and compare the monitored parameters with bag-valve ventilation.

The authors demonstrated similar rates of return for spontaneous circulation and arterial oxygen saturation; with statistically significant variations in tidal volume, CTE_2,_ and peak inspiratory flow, with lower dynamic pulmonary compliance after WHR in the bag-valve group. The article concludes that *VLP2000E* ventilation is feasible during CPR and equivalent to bag-valve ventilation in terms of WHR rates and arterial oxygen saturation, but with a better profile of respiratory parameters and lower airway pressure and tidal volume.

Considering future and different scenarios such as pre-hospital environments or even emergency rooms, devices with this performance can optimize ventilation capacity during CPR, reducing the need for additional team members focused on ventilation and allowing greater focus of attention to chest compressions, without the loss of quality of ventilation and oxygenation. Cardiopulmonary resuscitation studies are very welcome in the Brazilian scientific literature, and the translation of preclinical studies to phase III studies for adequate evaluation of findings confers the next important step, similar to feedback devices and chest compression, to effectively recompose the quality of ventilation with lower impacts on lung injury and post-CRP periods.
